# Crystal structure of hexa­kis­(*N*,*N*-di­methyl­form­amide-κ*O*)iron(III) μ-chlorido-bis­(tri­chlorido­cadmium)

**DOI:** 10.1107/S2056989021009580

**Published:** 2021-09-21

**Authors:** Olga Yu. Vassilyeva, Vladimir N. Kokozay, Svitlana Petrusenko, Alexandre N. Sobolev

**Affiliations:** a Department of Chemistry, Taras Shevchenko National University of Kyiv, 64/13 Volodymyrska Street, Kyiv 01601, Ukraine; b School of Molecular Sciences, M310, University of Western Australia, 35 Stirling Highway, Perth, 6009, W.A., Australia

**Keywords:** crystal structure, Fe^III^, ionic salt, *N*,*N*-di­methyl­formamide, polychloride dicadmium anion

## Abstract

The title compound, [Fe(C_3_H_7_NO)_6_][Cd_2_Cl_7_], crystallizes in the trigonal space group *R*  and is assembled from discrete [Fe(DMF)_6_]^3+^ cations (DMF = *N*,*N*-di­methyl­formamide) and [Cd_2_Cl_7_]^3−^ anions. In the cation, the iron(III) atom, located on a special position of  site symmetry, is coordinated by six oxygen atoms from DMF ligands with all Fe—O distances being equal [2.0072 (16) Å]. A slight distortion of the octa­hedral environment of the metal comes from the *cis* O—Fe—O angles deviating from the ideal value of 90° [86.85 (7) and 93.16 (7)°] whilst all the *trans* angles are strictly 180°. The central Cl atom of the [Cd_2_Cl_7_]^3−^ anion is also located on a special position of  site symmetry and bridges two corner sharing, tetra­hedrally coordinated Cd^II^ atoms. The two Cd atoms and the central Cl atom are colinear. The two sets of terminal chloride ligands on either side of the dumbbell-like anion are rotated relative to each other by 30°. In the crystal, the cations and anions, stacked one above the other along the *c*-axis direction, are held in place principally by electrostatic inter­actions. There are also C—H⋯Cl and C—H⋯O inter­actions, but these are rather weak. Of the six crystal structures reported to date for ionic salts of [Fe(DMF)_6_]^*n*+^ cations (*n* = 2, 3), five contain Fe^II^ ions. The title compound is the second example of a stable compound containing the [Fe(DMF)_6_]^3+^ cation. The existence of both [Fe(DMF)_6_]^2+^ and [Fe(DMF)_6_]^3+^ cations shows that the DMF ligand coordination sphere can accommodate changes in the charge and spin states of the metal centre.

## Abstract 2

Octa­hedral [Fe(DMF)_6_]^3+^ and tetra­hedral-based [Cd_2_Cl_7_]^3−^ ions stack alternately along the *c*-axis direction in this rare example of a [Fe(DMF)_6_]^3+^-containing ionic salt.

## Chemical context   

In our ongoing research into the new functions and applications of coordination compounds with Schiff-base ligands, we have utilized a synthetic scheme involving a zerovalent metal as the source of metal ions, together with another metal salt, in order to prepare new heterometallic complexes (Kokozay *et al.*, 2018 ▸; Vassilyeva *et al.*, 2018 ▸, 2021 ▸). In a typical procedure, the metal powder undergoes oxidative dissolution in air to generate metal ions that then inter­act with the second metal salt and pre-formed ligand. The condensation reaction between the Schiff-base precursors occurs *in situ* without isolation of the imine. Di­oxy­gen from the air is reduced to form a water mol­ecule with participation of protons donated by the imine, which is capable of deprotonation.

By using the above scheme, new homo- and heterometallic Co^III^, Co^III^/Zn^II^ and Co^III^/Cd^II^ complexes with a Schiff-base ligand derived from 2-hy­droxy-3-meth­oxy­benzaldehyde (*o*-vanillin) and the simple amine methyl­amine have been prepared (Nesterova *et al.*, 2018 ▸, 2019 ▸). Comparative studies of their catalytic behaviours in oxidation reactions of alkanes with H_2_O_2_ and *m*-chloro­per­oxy­benzoic acid were undertaken to elucidate the role of the second (inactive) metal centre (Cd) in the catalytic performance of the heterometallic compounds. Given the remarkable catalytic activity of the Schiff base Fe^III^ metal complexes mimicking the Fe-containing enzymes that oxidize alkanes in nature (Nesterov *et al.*, 2015 ▸), we decided to extend our work and replace the cobalt centre with iron in a heterometallic core supported by the above Schiff-base ligand.

To facilitate formation of the desired compound, an additional basic agent, *N*-phenyldi­ethano­lamine, was introduced following the previous successful participation of di­ethano­lamine in the formation of a mixed-ligand Schiff base Ni^II^/Zn^II^ dimer (Vassilyeva *et al.*, 2021 ▸). In the latter compound, the deprotonated amino­alcohol mol­ecules provide additional alkoxo-bridges between the metal centres. The use of amino­alcohol deprotonation in reactions employing zero­valent metals in the synthesis of heterometallics was established by a number of us several years ago (Vassilyeva *et al.*, 1997 ▸; Buvaylo *et al.*, 2005 ▸, 2012 ▸).
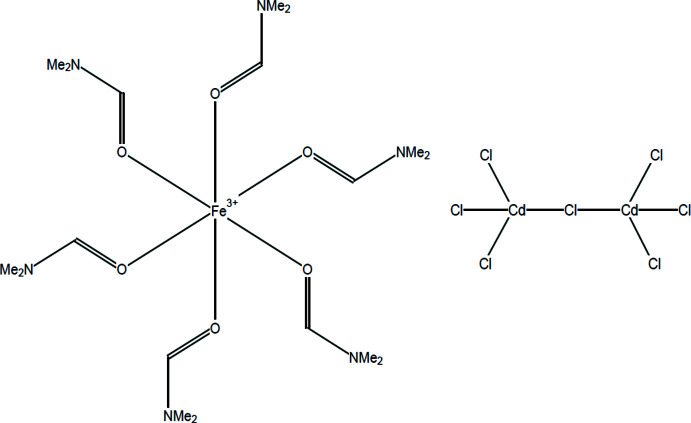


In the present work, the treatment of cadmium powder and FeCl_3_·6H_2_O with a solution of the *in situ*-formed Schiff base in open air worked a different way than expected and led to the isolation of the title compound, the mixed-metal ionic salt [Fe^III^(DMF)_6_][Cd_2_Cl_7_], (**1**), the identity of which was established by X-ray crystallography and confirmed by chemical analysis.

## Structural commentary   

**Figure 1:**
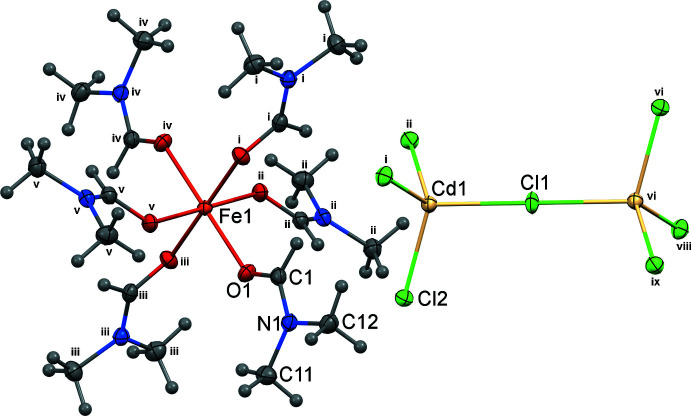
Mol­ecular structure and labelling of [Fe^III^(DMF)_6_][Cd_2_Cl_7_] (**1**) with displacement ellipsoids at the 50% probability level. [Symmetry codes: (i) −*y* + 1, *x* − *y* + 1, *z*; (ii) −*x* + *y*, −*x* + 1, *z*; (iii) *y* − 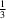, −*x* + *y* + 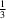, −*z* + 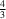; (iv) −*x* + 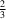, −*y* + 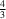, −*z* + 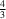; (v) *x* − *y* + 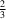, *x* + 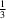, −*z* + 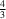; (vi) −*x* + 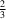, −*y* + 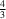, −*z* + 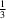; (viii) *y* − 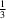, −*x* + *y* + 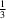, −*z* + 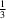; (ix) *x* − *y* + 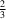, *x* + 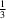, −*z* + 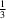.]

Compound (**1**), [Fe(C_3_H_7_NO)_6_][Cd_2_Cl_7_], crystallizes in the trigonal space group *R* 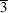 and is assembled from discrete [Fe(DMF)_6_]^3+^ cations (DMF = *N*,*N*-di­methyl­formamide) and [Cd_2_Cl_7_]^3−^ anions. In the cation, the iron(III) atom sits on a special position of 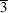 site symmetry and is coordinated by six oxygen atoms from the DMF ligands with all the Fe—O bond lengths being equal at 2.0072 (16) Å (Fig. 1 ▸, Table 1 ▸). The octa­hedral environment of the metal is slightly distorted as a result of the *cis* O1—Fe1—O1 angles deviating from the ideal value of 90° [86.85 (7) and 93.16 (7)°] while all the *trans* angles are strictly 180°. The central Cl atom of the [Cd_2_Cl_7_]^3−^ anion, Cl1, is also located on a special position of 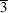 site symmetry and bridges two corner-sharing, tetra­hedrally coordinated Cd^II^ atoms. The two Cd atoms and the central Cl atom are colinear (Cd1—Cl1—Cd1^vi^ angle = 180°) and the bridging Cd1⋯Cd1^vi^ distance is 5.0752 (3) Å (Fig. 1 ▸). The two sets of terminal chloride ligands, Cl2, on either side of the dumbbell–like anion are rotated relative to each other by 30°. Around each Cd atom, the bridging Cd—Cl1 distance at 2.5377 (3) Å is 0.1 Å longer than that of the terminal Cd—Cl2 distance (2.4358 (5) Å) and the Cl2—Cd1—Cl1 and Cl2—Cd1—Cl2^i^ angles are 107.547 (14) and 111.325 (13)°, respectively, which are very close to the ideal value of 109°. The bond lengths and angles of the DMF ligands are similar to those found in [Fe(DMF)_6_](ClO_4_)_3_ (Houlton *et al.*, 2015 ▸).

## Supra­molecular features   

**Figure 2:**
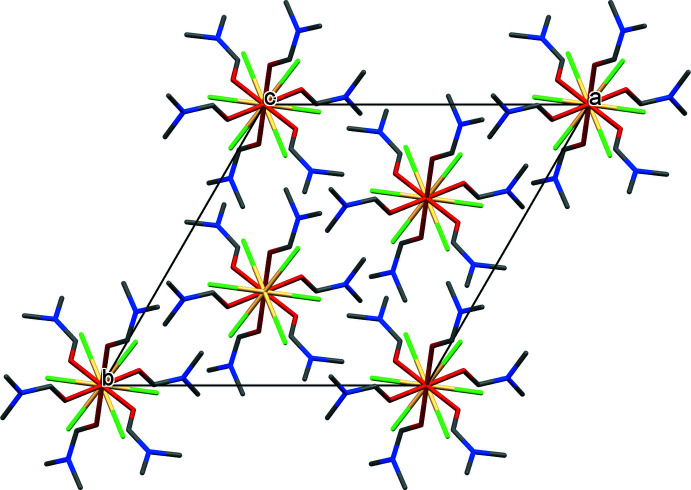
Crystal packing of (**1**) along the *c* axis showing stacks of cations and anions alternating in the *c*-axis direction. Hydrogen atoms are not shown.

In the crystal, the cations and anions are stacked one above the other along the *c*-axis direction (Fig. 2 ▸). Although classical hydrogen bonds are absent, several weak C—H⋯O and C—H⋯Cl inter­actions are detected in the structure [C1—H1⋯Cl2^i^, 3.772 (3) Å; C12—H123⋯Cl12^vii^, 3.783 (3) Å and C1—H1⋯O1^i^, 3.097 (3) Å]. The minimum H⋯O distance (H1⋯O^i^) between DMF mol­ecules within the same cation is 2.62 (3) Å and the shortest distance between Cl atoms of the anions and adjacent H atoms of DMF methyl groups (H123⋯Cl2^vii^) is 2.82 Å (Table 2 ▸), implying that the halide ions act as weak hydrogen-bond acceptors.

## Database survey   

A survey of the Cambridge Structural Database (CSD, Version 5.42, update May 2021; Groom *et al.*, 2016 ▸) reveals six ionic salts containing octa­hedral [Fe(DMF)_6_]^*n*+^ (*n* = 2, 3) cations. Five of the structures contain Fe^II^ ions, which crystallize in the presence of the counter-anions [FeCl_4_]^2−^ (CALMOS01; Cheaib *et al.*, 2013 ▸), [FeCl_2_S_4_W]^2−^(CUSNOT; Coucouvanis *et al.*, 1984 ▸), [Mo_2_S_6_]^2−^ (DEZMIF; Li *et al.*, 2007 ▸), [Fe_2_Cl_4_S_2_]^2−^ (VAMFIY; Müller *et al.*, 1989 ▸) and ClO_4_^−^ (GAZGET; Baumgartner, 1986 ▸). The only example to date containing the Fe^III^ cation, [Fe(DMF)_6_]^3+^, is found as the perchlorate salt (DMFAFE01; Houlton *et al.*, 2015 ▸).

In the pair of perchlorate salts, the Fe^II^ and Fe^III^ ions are easily distinguishable by their dissimilar Fe—O bond distances that vary in the ranges 2.08–2.11 and 1.9869 (15)–1.9985 (14) Å for [Fe(DMF)_6_](ClO_4_)_2_ (GAZGET) and [Fe(DMF)_6_](ClO_4_)_3_ (DMFAFE01), respectively. Both Fe-based octa­hedra are only slightly distorted with *cis* bond angles in the ranges 86.3–93.7° (GAZGET) and 88.57 (6)–91.43 (6)° (DMFAFE01), while all the *trans* angles are equal to the ideal value of 180°. The geometric parameters of the [Fe(DMF)_6_]^3+^ cation in the title compound, (**1**), are very close to those found in [Fe(DMF)_6_](ClO_4_)_3_ (DMFAFE01) with slight differences arising due to the different counter-anions present. The existence of both [Fe(DMF)_6_]^2+^ and [Fe(DMF)_6_]^3+^ cations shows that the DMF ligand coordination sphere can accommodate changes in the charge and spin states of the metal centre.

Considering the anion found in (**1**), there are six more examples of [Cd_2_Cl_7_]^3−^ anions in the CSD [LOVLUF (Chen *et al.*, 2014 ▸); MANBIP and MANCAI (Shen *et al.*, 2017 ▸); NIZXUR (Zhou *et al.*, 2014 ▸); WEYLUJ (Sharma *et al.*, 2012 ▸) and YAYFIQ (Cui *et al.*, 2017 ▸)] with different degrees of distorted tetra­hedral geometry around the Cd atoms and a Cd—Cl—Cd angle ranging from 103.92 (4)° in [Co(phen)_3_][Cd_2_Cl_7_]·3H_2_O (WEYLUJ) to 180° in (**1**). The Cd⋯Cd distance of 3.9983 (5) Å in the ‘bent’ structure is significantly lower than that found in (**1**) [5.0752 (3) Å], showing conformational flexibility of the polychloride dicadmium anion to achieve shape complementarity to the counter-cation.

## Synthesis and crystallization   

2-Hy­droxy-3-meth­oxy-benzaldehyde (0.3 g, 2 mmol) was stirred magnetically with CH_3_NH_2_·HCl (0.14 g, 2 mmol) and *N*-phenyldi­ethano­lamine (0.36 g, 2 mmol) in methanol (20 mL) in a 50 mL conical flask at 303 K for 20 min. A fine Cd powder (0.11 g, 1 mmol) and dry FeCl_3_·6H_2_O (0.27 g, 1 mmol) were introduced to the flask, and the mixture was kept stirring at 333 K to achieve dissolution of the zerovalent metal (1 h). The resulting dark blue–green solution was then filtered and allowed to evaporate at room temperature. After a week, the solution was diluted with DMF (7 mL) since it was thickening and filtered again. Dark-green octa­hedral crystals of (**1**) formed over two months after successive addition of Pr^i^OH (4 mL) and diethyl ether (4 mL) in several portions. The crystals were filtered off, washed with diethyl ether and finally dried in air. Yield (based on Fe): 0.13 g (64%). Analysis calculated for C_18_H_42_FeN_6_O_6_Cd_2_Cl_7_ (967.37): C 22.35, H 4.38, N 8.69%. Found: C 22.86, H 4.30, C 8.36%.

## Refinement   

Crystal data, data collection and structure refinement details are summarized in Table 3 ▸. Anisotropic displacement parameters were refined for all non-hydrogen atoms. All the carbon-bound hydrogen atoms were placed in calculated positions and refined using a riding model with isotropic displacement parameters based on those of the parent atom [C—H = 0.95 Å, *U*_iso_(H) = 1.2*U*_eq_(C) for CH and C—H = 0.98 Å, *U*_iso_(H) = 1.5*U*_eq_(C) for CH_3_].

## Supplementary Material

Crystal structure: contains datablock(s) I. DOI: 10.1107/S2056989021009580/cq2046sup1.cif

Structure factors: contains datablock(s) I. DOI: 10.1107/S2056989021009580/cq2046Isup2.hkl

CCDC reference: 2110079

Additional supporting information:  crystallographic information; 3D view; checkCIF report

## Figures and Tables

**Table 1:** Selected geometric parameters (Å, °)

Cd1—Cl2	2.4358 (5)	Fe1—O1	2.0072 (16)
Cd1—Cl1	2.5377 (3)		
			
Cl2^i^—Cd1—Cl2	111.325 (13)	O1^ii^—Fe1—O1	93.15 (7)
Cl2—Cd1—Cl1	107.547 (14)	O1^iii^—Fe1—O1	86.84 (7)

Symmetry codes: (i) -y+1, x-y+1, z; (ii) -x+y, -x+1, z; (iii) y-{\script{1\over 3}}, -x+y+{\script{1\over 3}}, -z+{\script{4\over 3}}.

**Table 2:** Hydrogen-bond geometry (Å, °)

*D*—H⋯*A*	*D*—H	H⋯*A*	*D*⋯*A*	*D*—H⋯*A*
C12—H123⋯Cl2^vii^	0.98	2.82	3.783 (3)	167
C1—H1⋯Cl2^i^	0.97 (3)	2.86 (3)	3.772 (3)	158 (2)
C1—H1⋯O1^i^	0.97 (3)	2.62 (3)	3.097 (3)	111 (2)
C12—H122⋯Cl2^i^	0.98	2.94	3.861 (3)	157

Symmetry codes: (i) -y+1, x-y+1, z; (vii) -x+{\script{4\over 3}}, -y+{\script{5\over 3}}, -z+{\script{2\over 3}}.

**Table 3:** Experimental details

Crystal data	
Chemical formula	[Fe(C_3_H_7_NO)_6_][Cd_2_Cl_7_]
*M*_r_	967.37
Crystal system, space group	Trigonal, *R*\overline{3}
Temperature (K)	100
*a*, *c* (Å)	13.7143 (2), 16.1312 (2)
*V* (Å^3^)	2627.51 (5)
*Z*	3
Radiation type	Cu *K*α
μ (mm^−1^)	18.18
Crystal size (mm)	0.06 × 0.05 × 0.05

Data collection	
Diffractometer	Oxford Diffraction Gemini-R Ultra, Ruby CCD
Absorption correction	Multi-scan (*CrysAlis PRO*; Rigaku OD, 2015 ▸)
*T*_min_, *T*_max_	0.760, 1.0
No. of measured, independent and observed [*I* > 2σ(*I*)] reflections	16143, 1051, 980
*R*_int_	0.038
(sin θ/λ)_max_ (Å^−1^)	0.598

Refinement	
*R*[*F*^2^ > 2σ(*F* ^2^)], *wR*(*F* ^2^), *S*	0.020, 0.057, 1.00
No. of reflections	1051
No. of parameters	67
H-atom treatment	H atoms treated by a mixture of independent and constrained refinement
Δρ_max_, Δρ_min_ (e Å^−3^)	0.79, −0.33

Computer programs: *CrysAlis PRO* (Rigaku OD, 2015 ▸), *SHELXT* (Sheldrick, 2015*a* ▸), *SHELXL2014/7* (Sheldrick, 2015*b* ▸) and *Mercury* (Macrae *et al.*, 2020 ▸).

## supplementary crystallographic information

### Crystal data

**Table:**

[Fe(C_3_H_7_NO)_6_][Cd_2_Cl_7_]	*D*_x_ = 1.834 Mg m^−^^3^
*M**_r_* = 967.37	Cu *K*α radiation, λ = 1.54178 Å
Trigonal, *R*3	Cell parameters from 9616 reflections
*a* = 13.7143 (2) Å	θ = 4.6–67.0°
*c* = 16.1312 (2) Å	µ = 18.18 mm^−^^1^
*V* = 2627.51 (5) Å^3^	*T* = 100 K
*Z* = 3	Octahedral, dark green
*F*(000) = 1443	0.06 × 0.05 × 0.05 mm

### Data collection

**Table:**

Oxford Diffraction Gemini-R Ultra, Ruby CCD diffractometer	1051 independent reflections
Radiation source: Enhance (Cu) X-ray Source	980 reflections with *I* > 2σ(*I*)
Mirror monochromator	*R*_int_ = 0.038
Detector resolution: 10.4738 pixels mm^-1^	θ_max_ = 67.2°, θ_min_ = 4.6°
ω scans	*h* = −16→15
Absorption correction: multi-scan (CrysAlisPro; Rigaku OD, 2015)	*k* = −16→16
*T*_min_ = 0.760, *T*_max_ = 1.0	*l* = −19→19
16143 measured reflections	

### Refinement

**Table:**

Refinement on *F*^2^	0 restraints
Least-squares matrix: full	Hydrogen site location: mixed
*R*[*F*^2^ > 2σ(*F*^2^)] = 0.020	H atoms treated by a mixture of independent and constrained refinement
*wR*(*F*^2^) = 0.057	*w* = 1/[σ^2^(*F*_o_^2^) + (0.0422*P*)^2^ + 4.220*P*] where *P* = (*F*_o_^2^ + 2*F*_c_^2^)/3
*S* = 1.00	(Δ/σ)_max_ = 0.001
1051 reflections	Δρ_max_ = 0.79 e Å^−^^3^
67 parameters	Δρ_min_ = −0.33 e Å^−^^3^

### Special details

**Table:**

Geometry. All esds (except the esd in the dihedral angle between two l.s. planes) are estimated using the full covariance matrix. The cell esds are taken into account individually in the estimation of esds in distances, angles and torsion angles; correlations between esds in cell parameters are only used when they are defined by crystal symmetry. An approximate (isotropic) treatment of cell esds is used for estimating esds involving l.s. planes.

### Fractional atomic coordinates and isotropic or equivalent isotropic displacement parameters (Å^2^)

**Table:**

	*x*	*y*	*z*	*U*_iso_*/*U*_eq_	
Cd1	0.3333	0.6667	0.32398 (2)	0.01664 (12)	
Fe1	0.3333	0.6667	0.6667	0.0139 (2)	
O1	0.47367 (14)	0.75401 (14)	0.59890 (10)	0.0200 (4)	
N1	0.59569 (16)	0.90250 (17)	0.52091 (12)	0.0187 (4)	
C1	0.4941 (2)	0.8248 (2)	0.54225 (15)	0.0191 (5)	
H1	0.431 (2)	0.819 (2)	0.5111 (17)	0.023*	
C11	0.6967 (2)	0.9151 (2)	0.56042 (17)	0.0257 (6)	
H113	0.7392	0.9902	0.5852	0.039*	
H111	0.7435	0.9055	0.5189	0.039*	
H112	0.6754	0.8579	0.6038	0.039*	
C12	0.6127 (2)	0.9772 (2)	0.45107 (16)	0.0244 (5)	
H123	0.6547	0.9645	0.4073	0.037*	
H121	0.6553	1.0557	0.4696	0.037*	
H122	0.5394	0.9614	0.4295	0.037*	
Cl1	0.3333	0.6667	0.1667	0.0233 (3)	
Cl2	0.51428 (4)	0.69293 (5)	0.36951 (3)	0.02102 (16)	

### Atomic displacement parameters (Å^2^)

**Table:**

	*U*^11^	*U*^22^	*U*^33^	*U*^12^	*U*^13^	*U*^23^
Cd1	0.01759 (14)	0.01759 (14)	0.01476 (17)	0.00879 (7)	0.000	0.000
Fe1	0.0141 (3)	0.0141 (3)	0.0136 (4)	0.00706 (14)	0.000	0.000
O1	0.0207 (8)	0.0213 (8)	0.0183 (8)	0.0107 (7)	0.0051 (6)	0.0042 (7)
N1	0.0201 (10)	0.0195 (10)	0.0164 (10)	0.0097 (8)	0.0014 (8)	−0.0016 (8)
C1	0.0214 (12)	0.0210 (12)	0.0159 (11)	0.0114 (10)	−0.0012 (9)	−0.0029 (10)
C11	0.0225 (13)	0.0261 (13)	0.0290 (14)	0.0126 (11)	−0.0022 (10)	−0.0020 (11)
C12	0.0248 (13)	0.0267 (13)	0.0217 (13)	0.0129 (11)	0.0041 (10)	0.0036 (10)
Cl1	0.0300 (5)	0.0300 (5)	0.0100 (6)	0.0150 (2)	0.000	0.000
Cl2	0.0191 (3)	0.0249 (3)	0.0201 (3)	0.0119 (2)	−0.0019 (2)	−0.0020 (2)

### Geometric parameters (Å, º)

**Table:**

Cd1—Cl2^i^	2.4358 (5)	N1—C11	1.455 (3)
Cd1—Cl2^ii^	2.4358 (5)	N1—C12	1.461 (3)
Cd1—Cl2	2.4358 (5)	C1—H1	0.97 (3)
Cd1—Cl1	2.5377 (3)	C11—H113	0.9800
Fe1—O1^iii^	2.0071 (16)	C11—H111	0.9800
Fe1—O1^ii^	2.0072 (16)	C11—H112	0.9800
Fe1—O1^i^	2.0072 (16)	C12—H123	0.9800
Fe1—O1	2.0072 (16)	C12—H121	0.9800
Fe1—O1^iv^	2.0072 (16)	C12—H122	0.9800
Fe1—O1^v^	2.0072 (16)	Cl1—Cd1^vi^	2.5376 (3)
O1—C1	1.258 (3)	Cd1—Cd1^vi^	5.0752 (3)
N1—C1	1.307 (3)		
			
Cl2^i^—Cd1—Cl2^ii^	111.323 (13)	C1—O1—Fe1	129.31 (16)
Cl2^i^—Cd1—Cl2	111.325 (13)	C1—N1—C11	123.0 (2)
Cl2^ii^—Cd1—Cl2	111.324 (13)	C1—N1—C12	120.4 (2)
Cl2^i^—Cd1—Cl1	107.547 (14)	C11—N1—C12	116.51 (19)
Cl2^ii^—Cd1—Cl1	107.549 (14)	O1—C1—N1	123.8 (2)
Cl2—Cd1—Cl1	107.547 (14)	O1—C1—H1	117.8 (17)
O1^iii^—Fe1—O1^ii^	86.85 (7)	N1—C1—H1	118.4 (17)
O1^iii^—Fe1—O1^i^	180.0	N1—C11—H113	109.5
O1^ii^—Fe1—O1^i^	93.16 (7)	N1—C11—H111	109.5
O1^ii^—Fe1—O1	93.15 (7)	H113—C11—H111	109.5
O1^iii^—Fe1—O1	86.84 (7)	N1—C11—H112	109.5
O1^i^—Fe1—O1	93.15 (7)	H113—C11—H112	109.5
O1^iii^—Fe1—O1^iv^	93.16 (7)	H111—C11—H112	109.5
O1^ii^—Fe1—O1^iv^	86.85 (7)	N1—C12—H123	109.5
O1^i^—Fe1—O1^iv^	86.84 (7)	N1—C12—H121	109.5
O1—Fe1—O1^iv^	180.0	H123—C12—H121	109.5
O1^iii^—Fe1—O1^v^	93.16 (7)	N1—C12—H122	109.5
O1^ii^—Fe1—O1^v^	180.00 (8)	H123—C12—H122	109.5
O1^i^—Fe1—O1^v^	86.84 (7)	H121—C12—H122	109.5
O1—Fe1—O1^v^	86.85 (7)	Cd1^vi^—Cl1—Cd1	180.0
O1^iv^—Fe1—O1^v^	93.15 (7)		
			
Fe1—O1—C1—N1	156.61 (17)	C12—N1—C1—O1	176.2 (2)
C11—N1—C1—O1	0.4 (4)		

Symmetry codes: (i) −*y*+1, *x*−*y*+1, *z*; (ii) −*x*+*y*, −*x*+1, *z*; (iii) *y*−1/3, −*x*+*y*+1/3, −*z*+4/3; (iv) −*x*+2/3, −*y*+4/3, −*z*+4/3; (v) *x*−*y*+2/3, *x*+1/3, −*z*+4/3; (vi) −*x*+2/3, −*y*+4/3, −*z*+1/3.

### Hydrogen-bond geometry (Å, º)

**Table:**

*D*—H···*A*	*D*—H	H···*A*	*D*···*A*	*D*—H···*A*
C12—H123···Cl2^vii^	0.98	2.82	3.783 (3)	167
C1—H1···Cl2^i^	0.97 (3)	2.86 (3)	3.772 (3)	158 (2)
C1—H1···O1^i^	0.97 (3)	2.62 (3)	3.097 (3)	111 (2)
C12—H122···Cl2^i^	0.98	2.94	3.861 (3)	157

Symmetry codes: (i) −*y*+1, *x*−*y*+1, *z*; (vii) −*x*+4/3, −*y*+5/3, −*z*+2/3.
